# State of the art review on machine learning and artificial intelligence in the study of neonatal necrotizing enterocolitis

**DOI:** 10.3389/fped.2023.1182597

**Published:** 2023-05-26

**Authors:** Steven J. McElroy, Shiloh R. Lueschow

**Affiliations:** ^1^ Department of Pediatrics, University of California Davis, Sacramento, CA, United States; ^2^ Stead Family Department of Pediatrics, University of Iowa, Iowa City, IA, United States

**Keywords:** machine learning (ML), artificial intelligence (AI), necrotizing enterocolitis (NEC), biomarker discovery, disease modeling

## Abstract

Necrotizing Enterocolitis (NEC) is one of the leading causes of gastrointestinal emergency in preterm infants. Although NEC was formally described in the 1960's, there is still difficulty in diagnosis and ultimately treatment for NEC due in part to the multifactorial nature of the disease. Artificial intelligence (AI) and machine learning (ML) techniques have been applied by healthcare researchers over the past 30 years to better understand various diseases. Specifically, NEC researchers have used AI and ML to predict NEC diagnosis, NEC prognosis, discover biomarkers, and evaluate treatment strategies. In this review, we discuss AI and ML techniques, the current literature that has applied AI and ML to NEC, and some of the limitations in the field.

## 1. Introduction

Necrotizing enterocolitis (NEC) is a devastating, inflammatory disorder, which impacts mainly preterm infants and remains one of the most common gastrointestinal emergencies in the preterm infant population (1–6). In the United States alone, it is estimated that up to 9% of infants weighing less than 1,500 g at birth will develop NEC (7). The mortality rate from NEC is significant and has been reported up to 30%–50% depending on disease severity (1–6). Treatment strategies have remained limited, non-targeted, and have not changed significantly in decades (8). Although NEC was formally described in 1965 by Mizrahi et al., the specific causes have yet to be fully elucidated (1–6). To help clinicians with NEC diagnosis, Bell et al. published the first clinical staging system for NEC in 1978 that was designed to help clinicians know when to surgically intervene (9). Eight years later, Walsh and Kliegman published a modified version of Bell's staging system (9, 10). The Bell and Modified Bell staging systems have consistently been the most widely used clinical definitions and are considered the “gold standard” in the field. However, most researchers and clinicians now focus on Bell ≥2 and believe that Bell stage 1 or Modified Bell stage 1A and 1B are considered largely non-specific (11). This has led to the development of six newer definitions for NEC, which all propose to be superior at NEC diagnosis than the Bell and Modified Bell staging definitions (12–18).

While many discoveries are being made within the NEC field, which may help prevent or treat NEC in the future, there remain fundamental limitations that clinicians and scientists in the field face. First, there is no universal definition of NEC. As discussed in the last paragraph, there now exist multiple definitions of NEC and clinicians and scientists can choose the one that suits their purposes best. This can lead to differences in what clinicians diagnose as NEC at various institutions. An added challenge is that the etiology of NEC has yet to be fully understood. Many in the field believe that NEC is a multifaceted disease and is the common end point of several pathways and pathologies. This multifaceted nature of NEC has made biomarker discovery difficult. Despite the NEC field spending ample time, resources, and research focus attempting to discover better biomarkers to aid in better prevention and mitigation strategies, all biomarkers discovered thus far have been insufficient (19–21). Therefore, NEC as a disease has the potential to benefit greatly from artificial intelligence (AI) and machine learning (ML) (21–24). So far, AI has shown promise in identification and prediction of diseases, biomarker discovery, disease risk evaluation, and development of improved treatment plans for many diseases both for adults and neonates (25–31). While AI and ML studies applied to the healthcare setting have rapidly increased in recent years, most instances have been applied to common and more well-defined diseases such as sepsis or cancer and only a few published studies have applied AI and ML to NEC. This review will summarize basic concepts of AI and ML (Section 2), present and summarize the current published literature on AI and ML in NEC (Section 3), as well as describe some of the limitations and pitfalls of AI and ML (Section 4).

## 2. Artificial intelligence and machine learning in healthcare

Artificial intelligence (AI) has become an increasingly relevant topic in most aspects of life and has offered particular promise in the healthcare sector (32–35). Computers have the unique ability to quickly find patterns in massive datasets that would take the human eye and brain far longer to identify (33, 36). Because of this, as early as the 1980's it was thought that through machine learning (ML), AI had the potential to be used to identify disease patterns and ultimately improve healthcare. Although at the time the computational power and algorithms necessary for ML and AI to be used effectively were not available, within the past decade a massive amount of time and resources have been devoted into the advancement of computers, AI, and ML (33, 36, 37). These improvements have made applying AI and ML to electronic medical records (EMRs) and within the healthcare sector a real possibility (38). While many use AI and ML interchangeably, there is a distinction between the two. AI describes a machine/computer using math and logic to learn and problem solve similarly to how a human brain functions, which can be done with or without the use of ML (39–43). While ML, a subset of AI, is the use of mathematical modeling and algorithms which learn and improve without explicit instruction as more data is provided (39–43). To put more simply, ML is just one application of AI, but other types of AI also exist such as limited memory AI, which is used for the development of chatbots or giving cars the ability to drive autonomously (39–43).

**Figure 1:**
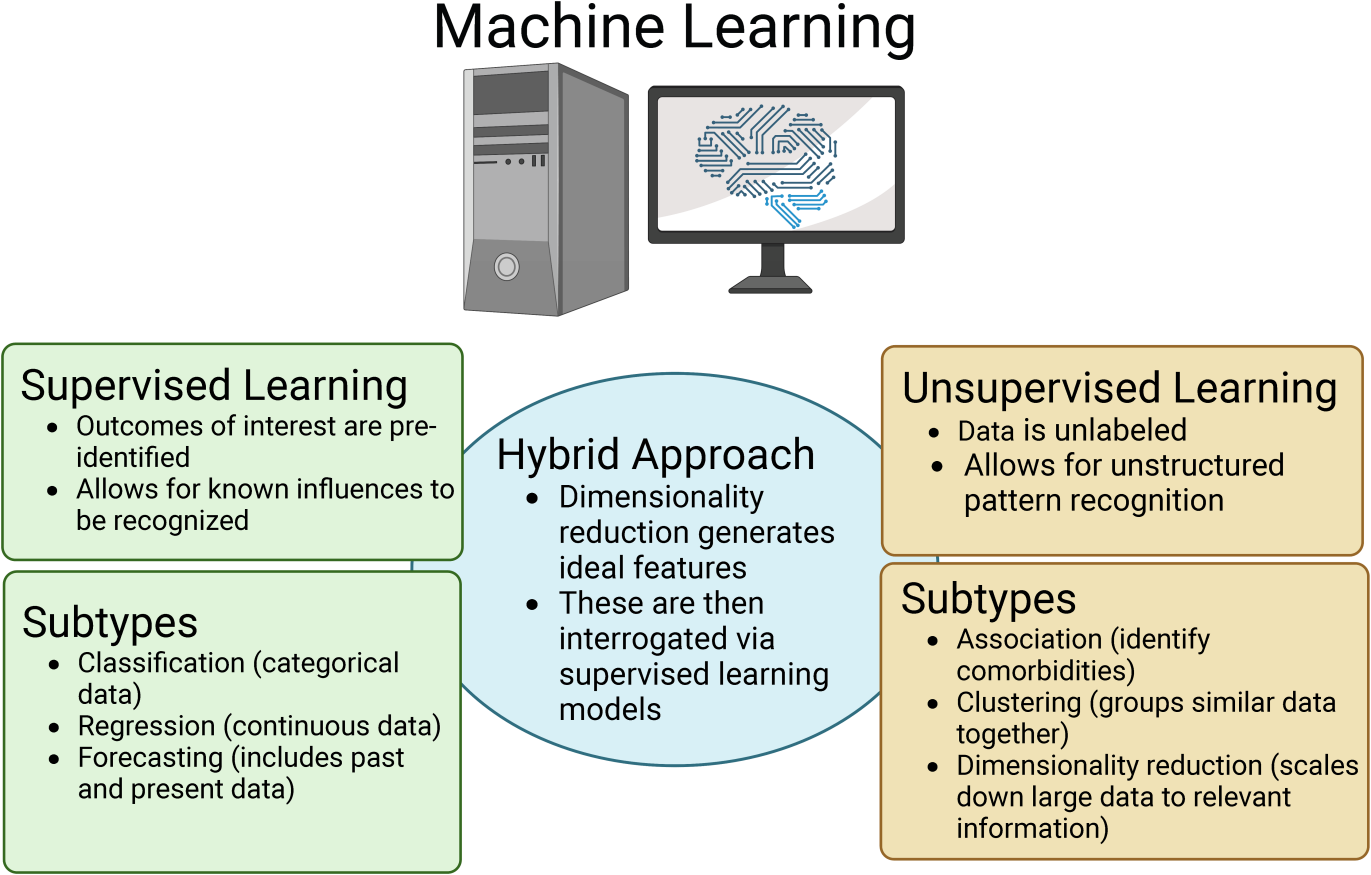
Overview of the three major types of machine learning (ML) that are applied in the healthcare setting as well as the respective subtypes. Figure created with Biorender.com.

Two main types of ML classifiers are used when AI is applied in the healthcare setting, which include supervised, or inductive classifiers, and unsupervised, which each have their own merits (Figure 1) (33, 36, 37). Supervised ML is used when the data has a labelled or identified outcome of interest. When using supervised ML in the neonatal healthcare setting, the dataset will contain features that are thought to influence an outcome (often EMR data including treatments, feeding types, gestational age, etcetera) as well as representation from the potential outcomes or labels of interest (disease vs. no disease; improvement, worsening, or no change following treatment; clinical disease scores; and so forth) (33, 36, 37). Within supervised ML, there are three subcategories depending on the data type including classification, regression, and forecasting (Figure 1). Classification supervised ML occurs when the output is categorical/discrete, whereas regression supervised ML uses continuous numerical values as output (33, 36, 37). The final type of supervised ML is forecasting, which is when both past and present data types are used as input to inform the model (33, 36, 37).

The other major type of ML is unsupervised ML, where unlabeled data is used as input and the ML model will identify patterns or structures within the data that would otherwise not be detectable to the human eye (33, 36, 37). Like supervised ML, unsupervised ML can also be divided into subcategories including association, clustering, and dimensionality reduction (Figure 1). Association models can be used to identify/predict comorbidities. In contrast, clustering models will group similar datasets together, but distinctly from others. For example, a clustering model would likely group patients with a disease condition together, but distinctly from patients without the disease (33, 36, 37). Finally, dimensionality reduction involves scaling down the data through the process of feature optimization. The process of dimensionality reduction is of particular importance when using EMRs and “omics” datasets because they house a wealth of information. However, because of the volume of data in these datasets, only a fraction of that information is useful when identifying/predicting disease (33, 36–38). Through dimensionality reduction, unsupervised ML models can identify what features best represent an outcome of interest vs. those that are superfluous. Thus, dimensionality reduction through feature optimization as well as feature engineering can be used for biomarker discovery. Dimensionality reduction can also aid in establishing a hybrid ML model (Figure 1). In this case, ideal features will be identified using an unsupervised ML model and then those features can be used as input into a supervised ML model to then predict a disease of interest. An additional approach to handling large data sets such as EMRs as well as “omics” data is using deep learning (DL). Deep learning can be used in the context of both supervised and unsupervised ML. DL uses higher complexity algorithms like neural networks and greater computational power to process large or high dimensionality datasets that some of the simpler ML models would have difficulty fitting (33, 36, 37).

Supervised and unsupervised ML models have similarities and differences in the required inputs. To create a supervised ML model, the dataset is first split into a training set, which will contain the majority or roughly 70%–80% of the data, and a test set, which will contain the remaining 20%–30% (33, 36, 37). If sufficient data is available, the 30% of data allocated for the test set can be split into both a test set (10%–15% of data) and a validation set (10%–15% of data). The validation set is utilized for parameter tuning within the various ML models, so that when the model reaches the testing/evaluation phase, the model is being tested on data it has never seen. Although ideal, if the overall dataset is relatively small and will not require a great deal of parameter tuning, the validation set may not be necessary (44). The training set will be provided to the ML algorithm of choice, ideally multiple different algorithms, and will include both the features as well as the labelled diagnoses/outcomes of interest. The ML algorithm will make a model based on the training data and then will apply the model created on the validation or test set. During the validation/testing stage, the model will use the features from the validation/test set that the model was originally trained on and attempt to predict the diagnosis/outcome. The model can then assess its own efficacy through accuracy scores (both training and test set accuracy), area under the receiver operator characteristics curve (AUROC), sensitivity, specificity, and other evaluation metrics (33, 36, 37, 44). The model developer can then fine tune the algorithm(s) parameters to improve upon the various evaluation metrics using the validation dataset. On the other hand, when using unsupervised ML models, there is no need to split the data into training and test sets because the data is unlabeled resulting in no way to formally evaluate the accuracy of the output. Instead, all the features of interest are used as input for each sample and then the algorithm(s) of choice is/are used to process the data before the model provides the desired output (33, 36, 37). While unsupervised machine learning models do not have the same degree of evaluation metrics, model developers can split data into a training and validation dataset. For unsupervised ML validation sets, it is important to have similar patterns and sample distribution as is present in the training set otherwise the ML model may have a false poor performance. If the ML model and the datasets were developed appropriately, similar output would be anticipated after running either set. For example, when using a clustering unsupervised ML model, samples would cluster similarly, and the same number of clusters would be found in both the training and validation set. Ultimately, while the input in supervised and unsupervised ML is different, using a validation set in both can help to ensure the model is being trained using the correct algorithm and is behaving in the way intended.

## 3. AI and ML in NEC

ML and AI studies and publications applied to the healthcare setting have rapidly increased in recent years, but most instances have been applied to common and more well-defined diseases such as sepsis or cancer (25–31). In comparison, relatively few studies have been published applying AI and ML to NEC (Table 1). While not formally described as ML, one of the earliest applications of computer science in the NEC literature came from the use of univariate and multivariate linear regressions, which was first documented in 1991 by Uauy et al. (61). In this publication, the modified Bell staging definition was used to distinguish suspected NEC (infants in stages IA and IB), proven NEC (infants in stage IIA), advanced NEC (infants in stage IIIA), and perforated NEC (infants in stage IIIB) (61). Demographic and clinical features of NEC were used as variables to determine statistical significance in the model distinguishing the various infant groups (61). Medical center, race, gender, birth weight, maternal hemorrhage, duration of ruptured membranes, and cesarean section were all identified as significant risk factors using this multicenter population and methodology (61). Since this publication, univariate and multivariate linear regressions continue to be utilized and seen in over 200 PubMed publications related to NEC to determine what risk factors are associated with NEC as was seen in the Uauy et al. publication or determining the prognosis of a patient with NEC based on treatment strategy. While linear regression is a form of classification ML, many debate whether univariate and multivariate linear regressions are considered true ML. Thus, these publications will not be discussed in detail in this review.

**Table 1:** Studies applying artificial intelligence (AI) and machine learning (ML) to NEC including a description of the cohort, type of ML, intended model purpose, and major findings from the model(s).

[Citation]	Cohort (#)	Type of ML Used	Purpose(s) of Model(s) Developed	Findings
Mueller et al. (45)	Multicenter; 197 patients (1) Control (130) (2) NEC (67)	Supervised ML [Artificial neural networks (ANN)]	(1) Determine risk factors for NEC	2 risk factors out of 57 were considered important in distinguishing NEC infants from controls 0.15 mean prediction error value from ANN model using 5 features
Sylvester et al. (46)	Multicenter; 485 patients (1) Medical NEC (345) (2) Surgical NEC (140) Multicenter; 65 patients	Supervised ML [Linear discriminant analysis (LDA)] Unsupervised ML (hierarchical clustering (UHC)	(1) Cluster analysis of urine biomarkers to see if peptide abundance will distinguish medical and surgical NEC infants (2) Distinguish medical NEC from surgical NEC infants using demographics and urine biomarker data	LDA model with clinical parameters: AUROC: 0.817 4 candidate urine peptides were identified in the clustering to best distinguish medical and surgical NEC Model with urine peptide biomarkers: AUROC: 0.86 LDA model with clinical features and urine peptide biomarkers: 100% correct outcome prediction
Doheny et al. (47)	Single Center; 70 preterm infants (1) Control (61) (2) NEC (9)	Supervised ML [Two step multiple logistic regression (LR)]	(1) Predict NEC based on the high frequency component of heart rate variability (HF-HRV)	Model sensitivity: 0.89; specificity: 0.87 Cutoff of 4.68 ms^2^ with NEC infants being below the cutoff was a non-invasive biomarker
Ji et al. (16)	Multicenter; 520 patients Clinical Concern for NEC (Bell stage IA-IIIA) (1) Confirmed/Medical NEC (344) (2) Surgical NEC (140) (3) Incomplete data (36)	Supervised ML [Hybrid generalized linear mixed effects models (GLMMs)] Hybrid (LDA)	(1) Objectively score NEC on a severity scale of I-III (2) Predict infants at low, intermediate or high risk for NEC progression	9 features were important for NEC severity scoring Severity score model agreement 100% for Bell stage 1, 94% for Bell stage 2; 83% for Bell stage 3 Outcome model AUROC: 0.84 2 features were important for outcome prediction
Irles et al. (48)	Single Center; 76 patients (1) Control (No NEC or IP) (27) (2) NEC without IP (23) (3) NEC with IP (26)	Supervised ML (ANN)	(1) Predict intestinal perforation (IP) associated with NEC from data available at birth (2) Predict IP associated with NEC from data available at birth and during hospitalization	Predicting with data available at birth *R*^2^: 0.976 Predicting with data available at birth and hospitalization *R*^2^: 0.98 11 features were important for prediction
Rusconi et al. (49)	Single Center; 96 patients (1) Control (67) (2) NEC Bell Stage ≥II (24) (3) NEC Bell Stage I (5)	Supervised ML (K nearest neighbors (KNN), Partial least squares (PLS), Random Forest (RF), Naïve Bayes (NB), Support Vector Machine (SVM)) Unsupervised ML (UHC)	(1) Predict NEC vs. non-NEC based on altered sphingolipid profiles from metabolomics data and demographic/clinical features	KNN model had the best performance: accuracy: 0.73 Clustering analysis suggested sphingolipid differences were important for distinguishing a subset, but not all NEC patients After including the sphingolipid clustering profile, better accuracy was achieved by the ML models: accuracy: 0.9–0.96
Olm et al. (50)	Single Center; 160 preterm infants (1) Control (126) (2) NEC (34)	Supervised ML [RF, Gradient Boosted Classifier (GBM)]	(1) Distinguish NEC from control infants based on clinical features and stool microbiome data (2) Determine the important features for model decision making	The GBM classifier performed better than RF: accuracy: 0.84 4 features categories were important for prediction
Hooven et al. (51)	Multicenter; 161 patients (1) Control (116) (2) NEC (45)	Unsupervised [Hierarchical Feature Engineering (HFE)] Supervised [Multi-layer neural network (MIL)]	(1) Predict risk for NEC based on serial stool microbiome taxonomy and demographic metadata	MIL: AUROC: 0.9 Prediction can take place over 24 h before disease onset
Gao et al. (52)	Single Center; 827 patients (1) Control (485) (2) NEC (342) Single Center; 379 NEC patients	Deep learning (DL) split attention networks, squeeze and excitation (SE) networks with/without the residual (Res) network (ResNest, SENet, SE-ResNet) Supervised ML (Light GBM)	(1) Predict NEC diagnosis based on 58 clinical features and radiomics data (2) Determine whether surgical intervention will be necessary using 49 clinical features and radiomics data (3) Determine what features were important for model decision making	LightGBM for NEC prediction: AUROC: 0.93; sensitivity: 0.94; specificity: 0.82 18 clinical features were important for prediction 9 clinical features were important for surgery prediction LightGBM model for surgery prediction: AUROC: 0.94; sensitivity: 0.95; specificity: 0.95
Pantalone et al. (53)	Single Center; 246 patients (1) Control (69) (2) Medical NEC (116) (3) Surgical NEC (61)	Supervised ML (LDA, RF, SVM)	(1) Distinguish the three groups based on clinical features and blood count data collected at birth, at baseline, at NEC diagnosis, and 3 days following antibiotic completion	Models had poor performance trying to classify all three together RF model performed the best distinguishing surgical NEC from controls: AUROC: 0.88; accuracy: 0.8; sensitivity: 0.8; specificity: 0.79 RF model performed the best distinguishing surgical NEC from medical NEC: AUROC: 0.76; accuracy: 0.67; sensitivity: 0.37; specificity: 0.82 4 features were important between the two models
Casaburi et al. (54)	Multicenter; 1,603 shotgun metagenomic datasets (1) 245 NEC positive (2) 1,358 non-NEC	Supervised ML (RF, GBM)	(1) Predict NEC based on taxonomic relative abundance data (2) Predict NEC or non-NEC based on clinical features and metagenomic taxonomy data (3) Calculate feature importance scores	RF model with species level taxonomy data and samples >29 weeks: test accuracy: 0.9 RF and GBM models with different PMA ≥ 29 or <29; balanced or unbalanced between NEC and non-NEC samples; stratified or unstratified: sensitivity: 0.24–0.92; specificity: 0.91–1.0 NEC associated Enterobacteriaceae spp. were the important features
Cho et al. (55)	Multicenter; 10,353 very low birth weight infants (1) Control (9,649) (2) NEC (704)	Supervised ML [LR, Decision Tree (DT), NB, RF, SVM, ANN]	(1) Predict risk for NEC based on 74 clinical features	LR and RF performed the best: accuracy: 0.93; AUROC: 0.73 and 0.72 respectively 10 clinical features were important for NEC prediction
Lin et al. (56)	Multicenter; 261 patients (1) Control (186) (2) NEC (75)	Supervised ML [Multiple instance neural network (MIL), RF]	(1) Predict NEC based on readily available clinical data and stool microbiome collected through onset of NEC or the first ∼60 days of life (2) Predict NEC based on stool microbiome instances the MIL model weighted as important	MIL: AUROC: 0.86–0.92; sensitivity: 0.86; specificity: 0.90 The microbiome data was important for the MIL model and based on the RF model, certain taxa (Firmicutes, Protobacteria, Enterobacteriaceae) drove the decision-making process The MIL model could predict NEC an average of 8.3 days prior to disease onset RF: AUROC: 0.79–0.86
Lueschow et al. (22)	Single Center; 219 patients (1) Control (117) (2) NEC (102)	Supervised ML [KNN, Simple neural network (SNN), NB, RF, SVM, DT]	(1) Predict NEC or non-NEC based on the features required for the NEC definitions (2) Determine important features based on the DT classifier (3) Develop a DT model using important features	DT model had the best performance: sensitivity: 0.83; specificity: 0.96; accuracy: 0.8; AUROC: 0.8 9 features were identified as important for the DT model decision making The most important feature definition DT model: sensitivity: 0.4; specificity: 0.77; accuracy: 0.62; AUROC: 0.62
Lure et al. (57)	Single Center; 40 patients undergoing surgical intervention (1) NEC (29) (2) Spontaneous intestinal perforation (SIP) (11)	Supervised ML [Ridge logistic regression (RLR), RF]	(1) Predict NEC or SIP	RLR: AUROC: 0.93; sensitivity: 0.89; specificity: 0.91 RF: AUROC: 0.98; sensitivity: 0.96; specificity: 0.96 4 variables were important for prediction with 3 associated with NEC and 1 with SIP
Qi et al. (58)	Single Center; 45 patients with NEC	Supervised ML (RF, SVM, LR)	(1) Predict whether NEC patients will need surgery or not based on clinical and radiomic features	(1) The RF model had the best performance with AUROC ranging from 0.68–0.8
Son et al. (59)	Multi-Center; 12,555 very low birth weight infants (1) Control (11, 703 Non-NEC) (2) NEC Non-IP (852) (3) NEC with IP (521) (4) SIP (208)	Supervised ML [ANN/multilayer perceptron (MLP), SVM (linear and radial), LR, KNN, DT, GBM (Light and extreme), RF]	(1) Predict NEC, NEC-IP or SIP (2) Predict NEC vs. Non-NEC then NEC-IP vs. SIP	The ANN/MLP had the best performance: AUROC: 0.81–0.87 depending on which condition it was predicting Applying the ANN/MLP model to a different dataset: AUROC: 0.67–1.0
Song et al. (60)	Single Center; 447 patients (1) Feeding intolerance (FI) (151) (2) NEC (296) Single Center; 296 NEC infants (1) Medical (205) (2) Surgical (91)	Supervised ML [Ridge regression and Q-learning strategy-based bee swarm optimization (RQBSO), SVM]	(1) Predict NEC vs. FI using 119 features (2) Predict the prognosis of NEC patients and whether they will require surgery using 119 features	NEC diagnosis compared to FI: AUROC: 0.94; accuracy: 0.91 7 features were notably important for NEC diagnosis NEC prognosis: AUROC: 0.92; accuracy: 0.84 5 Features were most important for NEC prognosis prediction

### 3.1. ML methods for NEC biomarker discovery

Biomarker discovery, particularly non-invasive biomarkers, and determining risk factors for NEC have been a topic of interest for researchers applying ML to NEC (Table 1). The first publication to formally apply ML to NEC was by Mueller et al. in 2009 (45). Using artificial neural networks (ANN), Mueller et al. found two risk factors from their set of 57 that were important for distinguishing NEC infants from controls including small for gestational age and being artificially ventilated (45). Additionally, the best scoring metric came from an ANN model using only five features (45). For biomarker discovery, Doheny et al. used the high frequency component of heart rate variability (HF-HRV) to predict NEC with high sensitivity and specificity in a multiple logistic regression model as infants that developed NEC had a much lower HF-HRV than infants that did not develop NEC (47). Pantalone et al. also used ML for biomarker discovery but chose to focus on the predictive ability of complete blood cell count (CBC) data at various time periods before NEC onset to distinguish between controls, patients with surgical NEC, and those with medical NEC (53). Their random forest (RF) model performed the best and while there were high performance scores in all metrics when distinguishing between surgical NEC and controls, the sensitivity was low when the RF model tried to classify surgical NEC compared to medical NEC (53). In both models, absolute bands at NEC and gestational age at birth were important contributors to the model (53). Cho et al. used six different supervised ML models to identify NEC based on 74 clinical features with the goal of understanding, which features may be important for NEC prediction. Two models, logistic regression (LR) and RF, had the best performance with high accuracy and decent AUROC scores (55). They also found 10 of the 74 features to be important for the RF model to distinguish NEC from controls (55).

Hooven et al., Lin et al., and Olm et al. all used stool microbiome data and demographic data to predict risk for NEC (50, 51, 56). In the publication by Hooven et al., following a dimensionality reduction approach through feature engineering, the stool microbiome and demographic data were used as input in a multi-layer neural network (MIL) model that had a high AUROC score (51). Importantly, the model Hooven et al. designed was able to predict NEC over 24 h before disease onset, but due to the complexity of the MIL model, it was difficult to interpret what features were required for the model to make decisions (51). As an extension of the Hooven et al. findings, Lin et al. used a similar hybrid approach with serial stool microbiome data, 10 clinical features, and the overall label of NEC vs. control (56). An unsupervised MIL model was used on each unlabeled stool sample within each patient's labeled set since it is unknown, which stool sample(s) within the set is/are NEC. The stool sample data was used to feed an ANN supervised ML model to predict NEC (56). The model had a high AUROC score and depended more on the microbiome data than it did on the clinical features (56). Interestingly, their model was able to predict NEC an average of 8.3 days before onset and using a RF model they found that certain taxa associated with NEC such as Firmicutes, Proteobacteria, and Enterobacteriaceae within the stool were important for NEC prediction (56). Olm et al. developed ML models using taxonomic data as well as other data that can be gleaned from microbiome data such as secondary metabolite profiles, metabolic pathways, and bacterial replication rates (50). Four feature categories from the original 2,119 features were considered important for prediction and their gradient boosted classifier (GBM) had the best performance in distinguishing NEC infants from controls (50). Casaburi et al. used machine learning to predict NEC vs. control from shotgun metagenomics data collected from several published studies (54). Their RF model had high accuracy and when testing the models under various conditions, it was found that specificity was high, but sensitivity varied greatly (54). Like Lin et al., it was found that NEC associated bacteria such as the Enterobacteriaceae species like *Klebsiella pneumoniae* and *Enterobacter cloacae* were important for the model decision making as well as *Staphylococcus aureus* (54).

Rather than stool microbiome data, Rusconi et al. used stool samples to generate metabolomic data to determine if there were usable biomarkers that could distinguish NEC from non-NEC infants (49). They found that sphingolipid profiles varied between NEC infants and non-NEC infants and used the respective profiles to develop a K nearest neighbors (KNN) model (49). After doing unsupervised ML hierarchical clustering, they determined that sphingolipids were only useful to distinguish a subset of patients, but after including the sphingolipid clustering profile with the other clinical features, much better ML accuracy scores were observed (49). Sylvester et al. used ML methods for biomarker discovery from urine peptides (46). First, unsupervised ML was used to cluster NEC infants with various potential biomarker profiles to distinguish surgical NEC infants from medical NEC infants (46). One cluster of peptides classified as fibrinogen A were most useful and when developing a linear discriminate analysis (LDA) model using both clinical parameters and urine peptide biomarkers, the model was able to correctly classify 100% of the infants as either surgical NEC or medical NEC, while the model using only clinical features was unable to classify 39% of the patients (46). Song et al. designed an algorithm with the intent of determining features that would be important to distinguish NEC diagnosis from feeding intolerance (FI) and predicting whether infants with NEC will require surgery (60). In their model distinguishing NEC from FI, seven features from their original set of 119 were important for diagnosis and their model achieved a high AUROC score (60). With a similar AUROC score, the model predicting NEC prognosis also had high performance and weighted five of the features as being most important for prediction (60).

### 3.2. ML used to predict NEC or NEC outcomes

Similarly, many publications have used ML to predict NEC. Ji et al. used generalized linear mixed effects models (GLMMs), on a dataset of 27 clinical features presented by the patients at first suspicion of NEC and historically had been associated with NEC prediction to determine NEC severity (16). Nine of the 27 features were important for the GLMMs to score NEC severity: “abdominal pain, pneumatosis intestinalis, portal venous gas, dilated bowel, air/fluid levels, thickened bowel walls, white blood cell count (WBC), % neutrophils, and neutrophil count” (16). Those nine significant features were used to develop a GLMM (supervised ML) and tested to determine whether it could provide similar scores to the clinician classifications (16). The model classified 100% of stage 1 infants correctly, 94% of stage 2% and 83% of stage 3 (16). Using an LDA algorithm (a dimensionality reduction approach for supervised classification ML), Ji et al. predicted infants at low, intermediate, or high risk for NEC progression (16). In this model, outcome score was most influenced by metabolic acidosis (pH) and portal venous gas (PVG) (16). While the AUROC score was relatively high, the model was unable to predict 18.9% of medical NEC and 57% of surgical NEC subjects and incorrectly predicted 0.6% of medical NEC and 21.4% of surgical NEC infants (16). ML models often struggle when data is missing, which is often the case when considering clinical data/EMRs (33, 36, 37, 44). A further interesting finding from Ji et al., was that their NEC outcome score model still had an AUROC score of roughly 80% when considering as few as five of their 27 features (16). While this groundbreaking study developed two relevant ML models applied to NEC severity diagnosis and prognosis respectively, there were limitations to the models including difficulty in risk stratification particularly of intermediate patients and disagreement in NEC score from the clinician classification in scores ≥2 (16).

### 3.3. ML methods to distinguish NEC with or without IP from spontaneous intestinal perforation (SIP)

While the publication by Ji et al. eliminated all infants with SIP three more recent publications by Irles et al., Lure et al., and Son et al. developed ML models involving SIP and IP (48, 57, 59). Irles et al. used back propagated ANN models on two datasets with one using 23 neonatal and maternal variables collected at birth and the other using 35 variables collected at birth as well as during hospitalization (48). Both models were able to effectively classify the infants (48). They went on to determine which variables were most informative for the model and found several variables associated with predicting IP including neonatal platelet and neutrophil counts, orotracheal intubation, birth weight, sex, arterial blood gas parameters, gestational age, use of fortifier, patent ductus arteriosus (PDA), maternal age, and maternal morbidity (48). Like Irles et al., Lure et al. found gestational age at birth to be associated with NEC as well as post menstrual age (PMA) prior to surgery, and pneumatosis, but found that pneumoperitoneum was associated with SIP (57). Additionally, their ML scoring metric (AUROC) was high with ridge logistic regression and RF models when radiographic findings were included as part of the input variables (57). Finally, Son et al. utilized several different ML algorithms to distinguish NEC infants with or without IP from those with SIP but had the most luck with ANN models/multilayer perceptron (MLP) (59). The first model distinguished between NEC, NEC with IP, and SIP and had reasonably high AUROC scores (59). In the second model, the first layer distinguished between NEC and NEC with IP, while the second layer distinguished between NEC with IP and SIP by utilizing data from the NEC infants from the first layer (59). They also used the models on a new dataset of patients and found an AUROC score of 0.67–1.0 depending on which condition was being predicted, with the highest AUROC score of 1.0 associated with predicting NEC-IP and 0.9 for predicting SIP (59).

### 3.4. ML methods to evaluate treatment options

Others have used ML to determine what NEC infants may benefit from a treatment such as surgery. For example, Qi et al. utilized LR, SVM, and RF models on a subset of radiographic and clinical features to predict whether surgery would be necessary for infants diagnosed with NEC (58). The RF model had a reasonable AUROC score using a feature engineered subset of 18 radiomic features and 14 clinical features from the original dataset of 79 features (58). Similarly, Gao et al. designed two different models using both clinical data as well as radiomics data (52). Using DL, Gao et al. scaled the radiomics data to use in a light GBM supervised ML classifier (52). The first model predicted NEC depending on 18 clinical features and the radiomics data with a high AUROC score (52). The second model was designed to predict whether surgery would be necessary for infants diagnosed with NEC (52). The second model placed importance on 9 of the clinical features and had also had a high AUROC score (52).

### 3.5. ML to evaluate currently available NEC definitions

Finally, in a recent publication from our lab, ML has been applied to evaluate the currently available definitions for NEC with the hope of developing a better definition (22). As mentioned earlier, there are now eight definitions for NEC including the original Bell and the modified Bell staging definitions and the more recent six definitions that have all been described within the last ten years (22). We found that the International Neonatal Consortium (INC) and 2 of 3 definitions had the best overall performance from the definitions and consistently outperformed the Bell and Modified Bell staging definitions (22). Additionally, we found nine features that were important for distinguishing NEC from non-NEC infants, but a model using only those nine features was not able to outperform previously described definitions (22).

## 4. Limitations and pitfalls for ML and AI

While ML and AI can be powerful tools, there are several pitfalls and limitations that must be taken into consideration when applying ML. First, as mentioned earlier, there is currently no universally accepted definition of NEC, and the Bell and Modified Bell staging definitions that are commonly used suffer from being non-specific to NEC until more severe stages of the disease have been reached. This means there can be discrepancies between what different institutions or even clinicians within an institution classify as NEC or the severity of NEC. Ultimately, this can lead to ML models being provided with subjective labels that may vary between institutions, which can make the model difficult to generalize to infants at other institutions. Along those lines, ML models can suffer from biases based on the input data, which can also make the models difficult to generalize (62). As an example, most studies discussed in this review were single center studies and some had as few as <100 patients. ML models often require 100 s–1,000 s of patients to be sufficiently trained and then additional patients to test/validate the model. Studies using few patients and only from a single center suffer from relatively homogenous populations. ML models trained on small and/or homogenous populations will have more difficulty properly classifying when heterogenous samples are added (62).

An added limitation is differences in EMRs that are often used as input for the clinical/demographic features. EMRs house a plethora of information, but there can be gaps in the data, subjective data, and differences in standard practices between institutions, which may limit its utility for ML purposes, or the generalizability of ML models developed (63). As discussed earlier, ML models struggle to cope with missing data. Thus, scientists developing ML models must make a choice between excluding patients, excluding certain features, imputing the data to fill the gaps, manually deciding for each gap the best way to fill in the feature, or some combination of these. Any decision made can have the potential of skewing the ML model. Other data processing may also be necessary to optimize a ML model's ability to appropriately classify such as scaling or normalizing certain features, which may impact generalizability when adding in different patients (63). Also, data points that are subjective and can vary between clinicians are challenging for a ML model to manage and can result in inaccurate predictions (63). Examples of features that may be subjective in nature are abdominal distension, lethargy, or radiologic findings as well as features that can have cutoffs that may vary between institutions such neutropenia, thrombocytopenia, or acidosis. Differences in standard practices between institutions can also skew the availability of EMR data points (63). For example, performing certain tests at birth may be standard at one hospital, but not at another, or the frequency at which certain tests are performed may vary between institutions which leads to gaps in the data available.

Finally, interpretability of developed ML models can be challenging (45, 64–66). One challenge in interpretability occurs when using feature engineering as it combines multiple different features into one. Hooven et al. used this approach to help scale down the metagenomics data, but they commented that although they knew the model depended on the metagenomics data since removing it resulted in lower performance metrics, it was hard to determine exactly what features within that dataset were important (51). Another challenge in interpretability that arises is when combining EMRs with omics data such as in Hooven et al., Lin et al., and Rusconi et al. (46, 54, 56). Omics datasets have massive numbers of features and require more complicated models to appropriately handle the data (30, 45). To understand more about complex models' decision-making process, separate ML models can be developed like Lin et al. who used a RF model to determine what taxonomic features from the microbiome data were important for the MIL model (54). Others used unsupervised ML through hierarchical clustering to narrow down the features that were used in the final model such as in Sylvester et al. and Rusconi et al. (46, 60). Requiring a secondary model to understand the model being developed adds another layer of complexity to the ML process and can make interpretability difficult for the eventual end users, the clinicians, who have varying levels of understanding of ML (65, 66).

## 5. Conclusions

While ML and AI have been utilized in the healthcare realm for decades with over 11,000 publications relating to cancer since 1985 and over 500 publications relating to sepsis since 1990, publications applying ML and AI to NEC have been far sparser. Nevertheless, the publications that have applied ML to NEC have covered a breadth of topics such as biomarker discovery, predicting NEC before onset, distinguishing NEC from other conditions, determining prognosis, or evaluating the current definitions of NEC. These studies have all provided promising data to aid in improving diagnosis and/or prognosis of infants with NEC, but there is plenty more that can be done in the future. As mentioned, many of the studies to date have been single center, used small patient sizes, and/or been rife with limitations. ML and AI models are only as good as the input they are provided (33). This reinforces the necessity to foster collaborations between researchers, clinicians, data scientists, biostatisticians, and bio-informaticists to provide future studies with clean, more widely generalizable datasets and overcome the many pitfalls and limitations that come with ML and AI. NEC as a disease has historically been difficult to diagnose and treat, but, if used effectively, ML and AI offer the potential to more quickly identify and diagnose NEC, help to predict the severity of the case, help optimize treatment strategies, and in summation provide an overall better prognosis for infants with NEC.
